# Supplementary figures and images for: Identification of microRNAs and their targets in inflorescences of an Ogura-type cytoplasmic male-sterile line and its maintainer fertile line of turnip (Brassica rapa ssp. rapifera) via high-throughput sequencing and degradome analysis
Source: PLoS One. 2020 Jul 30;15(7):e0236829. doi: 10.1371/journal.pone.0236829 (PMC7392268; doi:10.1371/journal.pone.0236829)

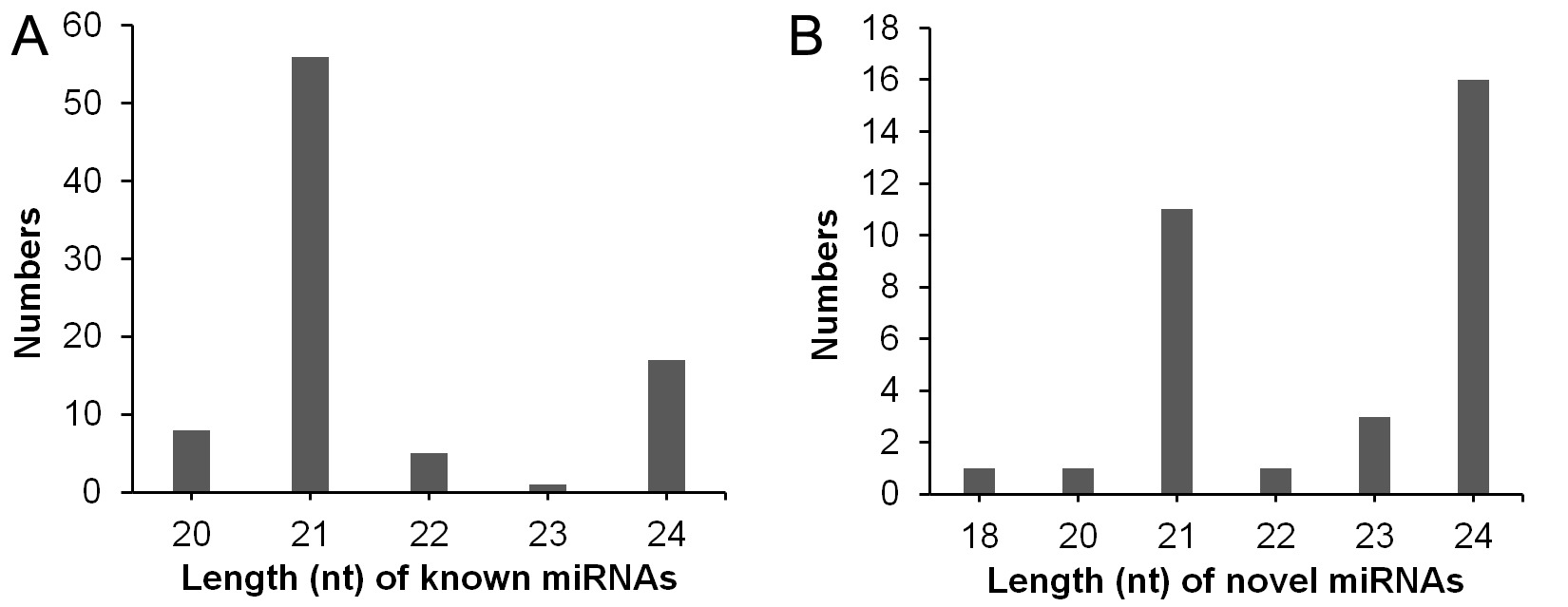

Supplement: S1 Fig — (A) Known miRNAs. (B) Novel miRNAs. (TIF) [file pone.0236829.s001.tif]

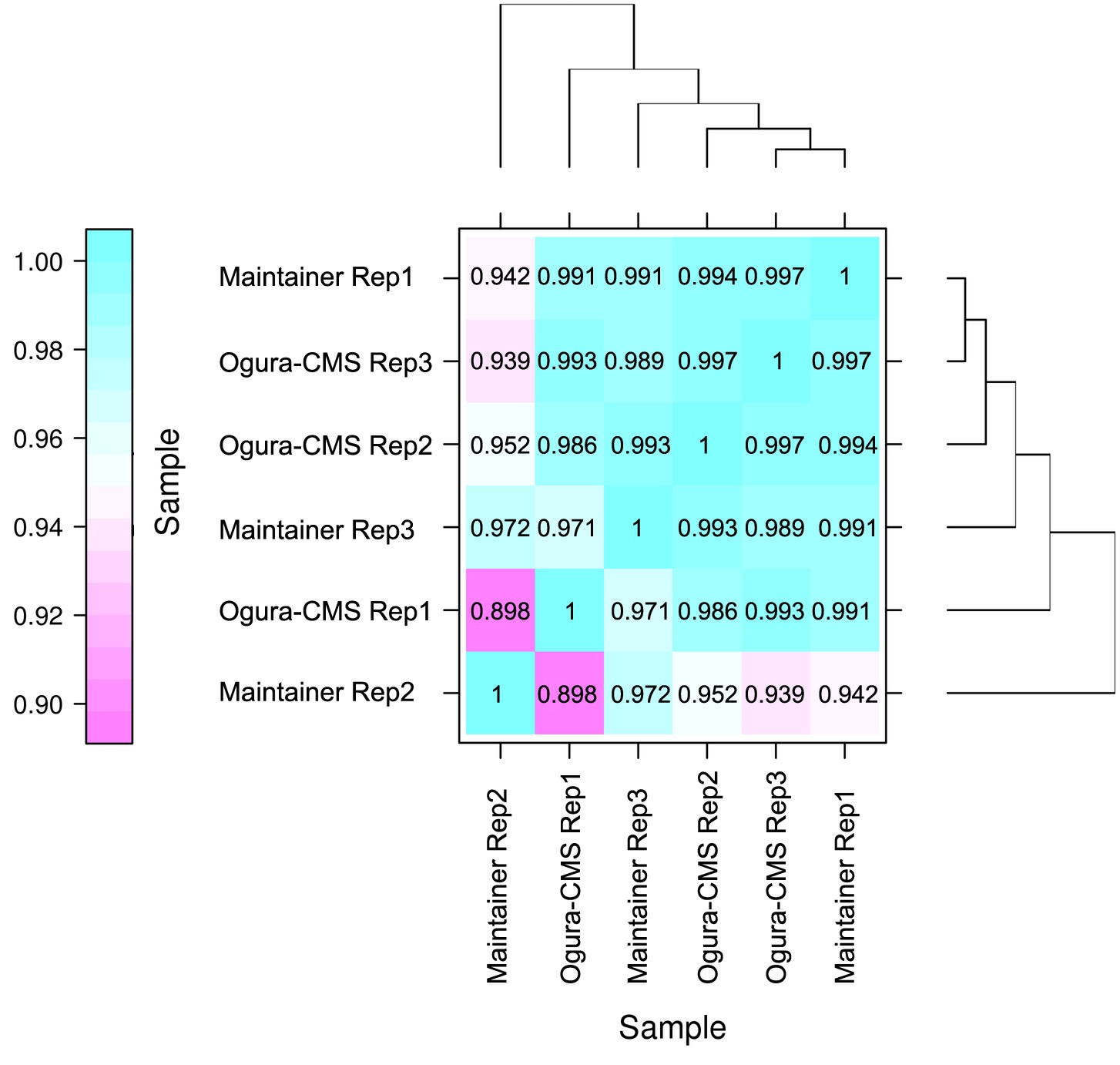

Supplement: S3 Fig — (TIF) [file pone.0236829.s003.tif]
